# Diffuse Hepatic and Spleen Uptake of Tc-99m MDP on Bone Scintigraphy Resembling Liver-Spleen Scintigraphy in a Patient of Plasma Cell Tumor

**DOI:** 10.1155/2014/264904

**Published:** 2014-03-25

**Authors:** Mohammad Reza Ravanbod, Reza Nemati, Hamid Javadi, Iraj Nabipour, Majid Assadi

**Affiliations:** ^1^Department of Oncology and Hematology, Bushehr Medical Center Hospital, Bushehr University of Medical Sciences, Tehran 7533934698, Iran; ^2^The Persian Gulf Nuclear Medicine Research Center, Bushehr University of Medical Sciences, Bushehr 7533934698, Iran; ^3^Golestan Research Center of Gastroenterology and Hepatology (GRCGH), Golestan University of Medical Sciences (GUOMS), Gorgan 4917765181, Iran; ^4^The Persian Gulf Tropical and Infectious Diseases Research Centre, Bushehr University of Medical Sciences, Bushehr 7533934698, Iran

## Abstract

The present case demonstrates a diffuse intense hepatic and, to a lesser degree, spleen, Tc-99m MDP uptake on a routine bone scintigraphy resembling liver-spleen imaging. A 49-year-old female with a history of anaplastic plasma cell tumor and suffering from bone pain was referred for bone scintigraphy to evaluate possible bone metastases. The bone scintigraphy showed diffuse hepatic and spleen uptake of Tc-99m MDP resembling liver-spleen imaging. Furthermore, bone uptake of Tc-99m MDP was significantly diminished and there were no abnormal foci throughout the skeleton. The bone scintigraphy of the present case of an anaplastic plasma cell tumor suggests the possible presence of amyloidosis.

## 1. Introduction

Amyloidosis is characterized by an abnormal extracellular deposition of amyloid in various tissues and organs of unknown cause [1, 2]. The pathogenesis of amyloid fibrils is associated with amino acid replacements in prefibrillar proteins and with protein instability [1]. Also, its clinical presentation, involving either single or multiple organs, results in different signs and symptoms [2].

Depicting the presence of amyloid protein in the tissue, mostly through invasive methods, is the primary way of diagnosing amyloidosis [3]. We describe a 49-year-old female with a history of anaplastic plasma cell tumor. Her bone scintigraphy with Tc-99m MDP suggested the possible presence of amyloidosis, by revealing intense diffuse tracer uptake in the liver and spleen.

## 2. Case Report

A 49-year-old female was referred for a bone scintigraphy for identification of possible bone metastasis. She complained of general bone pain. Her past history included a lytic lesion in the right mandible that had been treated with chemotherapy and radiotherapy. Several investigations included bone marrow aspiration cytology and immunohistochemistry (IHC), suggesting anaplastic plasma cell tumor. The serum electrolyte, calcium, and phosphate levels were increased. The serum creatinine level was also increased (Cr: 3.5 mg/dL).

**Figure 1:**
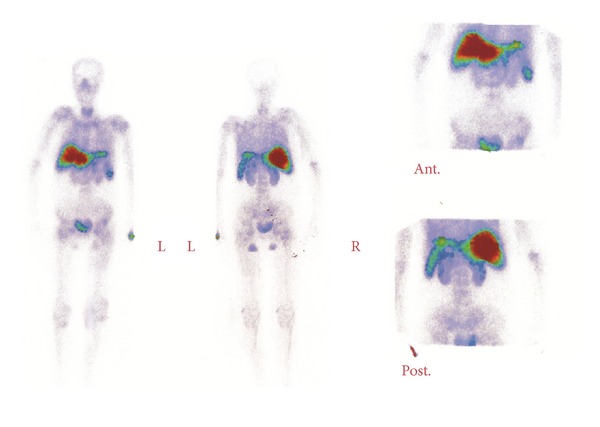
Anterior and posterior whole body bone scan, performed three hours after the intravenous administration of Tc-99m MDP, showed diffuse and intense tracer uptake in the liver and, to a lesser degree, in the spleen, without any remarkable abnormal activity in the skeleton. Decreased skeletal uptake was also observed.

Ultrasonography revealed that the patient had a mild splenomegaly. A bone scan revealed diffusely and intensely hepatic 99Tc-MDP uptake, and to a lesser degree in the spleen. Decreased skeletal uptake on bone scan was also observed (Figure 1). We suggested the possibility of immunoglobulin amyloidosis resulting from disturbed renal function in the base of the patient's underlying disease.

## 3. Discussion

Bone scintigraphy is frequently requested for identification of possible bone metastasis in patients with a primary tumor, even though it is generally indicated in multiple myeloma through osteolytic lesions that depict no 99m Tc-MDP tracer uptake [4]. A majority of such patients are referred for bone scintigraphy because their presentation mimics metastatic bone disease [4].

On the other hand, nonosseous uptake of bone-seeking radiopharmaceuticals is observed in different conditions and organs which are necessary to distinguish the pathophysiological causes [5, 6].

The first explanation is that it is a mistake in radiopharmaceutical preparation and the formation of Tc-99m colloid complex, resulting in abnormal distribution [7]. This explanation is ruled out in this patient, because bone scans of other patients were performed at the same time and did not demonstrate such abnormality.

The other explanations for hepatic activity are hepatic metastasis, necrosis, alcoholic liver disease, or history of liver transplantation. The patient's clinical manifestations, ultrasound of the abdomen, and other investigations were not consistent with such explanations [8–11].

In addition, diffuse hepatic uptake, accompanied by decreased skeletal uptake on bone scan, is normally noted after an intravenous injection of iron colloid solutions or methotrexate for treatment. The present case did not have such a history [12, 13].

Furthermore, extraosseous uptake on bone scan is observed in patients with renal failure as a result of a failure to excrete the radiopharmaceuticals through the kidneys [14]. Hypercalcemia and hyperparathyroidism resulting from chronic renal failure cause soft tissue microcalcification [14]. Likewise, splenic uptake has been seen in the bone scan of patients with sickle-cell disease as a result of splenic infarction and subsequent calcification [15]. Such explanations are not congruent with the present case.

Furthermore, extraosseous uptake on bone scan has been observed in amyloidosis [2]. Amyloidosis is characterized by a heterogeneous group of disorders related to abnormal extracellular protein deposits that can affect any organ and can lead to end-organ dysfunction [16].

Five types of amyloidosis have been defined based on the underlying disease: immunoglobulin amyloidosis, familial amyloidosis, senile-systemic amyloidosis, secondary amyloidosis, and hemodialysis-associated amyloidosis [2].

Few studies have suggested the use of scintigraphy as a screening test for amyloidosis. Different radiopharmaceuticals have been examined, including Ga-67 and Tc-99m sulfur colloid, with mixed results [17, 18]. Bone radionuclides have been shown to accumulate in different organs affected by amyloidosis, including the liver, skin, skeletal muscle, and myocardium [19, 20].

The precise mechanism by which bone-imaging radiopharmaceuticals accumulate within amyloids is not understood [2]. The extracellular deposition of amyloids may increase the local calcium level and subsequently enhance the affinity for bone-seeking agents at the sites of amyloid deposition. Nevertheless, when these radiopharmaceuticals accumulate considerably in soft tissue, the presence of amyloid deposition should be considered [2].

Molecular imaging using radiolabeled positron emission tomography (PET) tracers is now being employed for the diagnosis of amyloid *β* plaques in Alzheimer's patients. Such molecular imaging is currently into preclinical stages in diagnosis of cardiac and peripheral amyloidosis [16, 21].

Amyloidosis is frequently related to decreased renal function in patients with multiple myeloma, resulting from light chain deposition in various organs [14, 22]. In the present case, a diagnosis of amyloidosis with diminished renal function may provide an explanation for the abnormal distribution of tracer in the liver and spleen, even though histopathology was not preformed to confirm the presence of amyloidosis. Extraosseous soft tissue and visceral accumulation further raise the possibility of amyloidosis. The present case suggests that further investigation and a directed biopsy may be warranted.

## References

[1] Falk RH, Comenzo RL, Skinner M (1997). The systemic amyloidoses. *The New England Journal of Medicine*.

[2] Fard-Esfehani A, Assadi M (2005). Myocardial Tc-99m MDP uptake on the bone scintigraphy in the hemodialysis-associated amyliodosis (an incidental finding). *Alasbimn Journal*.

[3] Sipe JD, Benson MD, Buxbaum JN (2012). Amyloid fibril protein nomenclature: 2010 recommendations from the nomenclature committee of the International Society of Amyloidosis. *Amyloid*.

[4] Leonard RCF, Owen JP, Proctor SJ, Hamilton PJ (1981). Multiple myeloma: radiology or bone scanning?. *Clinical Radiology*.

[5] Gentili A, Miron SD, Bellon EM (1990). Nonosseous accumulation of bone-seeking radiopharmaceuticals. *Radiographics*.

[6] Sood A, Seam RK, Sethi S, Gupta M (2010). Diffuse hepatic and splenic Tc-99m MDP tracer uptake in case of multiple myeloma. *Indian Journal of Nuclear Medicine*.

[7] MacDonald J (2001). Idiopathic hepatic uptake of 99mTc methylene diphosphonate: a case report. *Journal of Nuclear Medicine Technology*.

[8] Shih W-J, Coupal J (1994). Diffuse and intense Tc-99m HMDP localization in the liver due to hypoxia secondary to respiratory failure. *Clinical Nuclear Medicine*.

[9] Kawamura E, Kawabe J, Hayashi T (2005). Splenic accumulation of Tc-99m HMDP in a patient with severe alcoholic cirrhosis of the liver. *Clinical Nuclear Medicine*.

[10] Munoz SJ, Nagelberg SB, Green PJ (1988). Ectopic soft tissue calcium deposition following liver transplantation. *Hepatology*.

[11] İlknur AK, İnci U (2008). Diffuse hepatic uptake of Tc-99m MDP on routine bone scan mimicking liver-spleen imaging: disseminated liver metastases from the gastric Cancer. *Turkish Journal of Nuclear Medicine*.

[12] Park CH, Kim HS, Shin HY, Kim HC (1997). Hepatic uptake of Tc-99m MDP on bone scintigraphy from intravenous iron therapy (Blutal). *Clinical Nuclear Medicine*.

[13] Lin KH, Shih BF, Tsao CH, Wu MC (2005). Diffuse liver uptake of technetium-99m-MDP bone scan due to hepatotoxicity secondary to methotrexate therapy. *Annals of Nuclear Medicine and Sciences*.

[14] Evans JC, Murphy M, Eyes B (2000). Extensive soft tissue uptake of99Tcm methylene diphosphonate in a patient with multiple myeloma. *British Journal of Radiology*.

[15] Goy W, Crowe WJ (1976). Splenic accumulation of (99m)Tc diphosphonate in a patient with sickle cell disease: case report. *Journal of Nuclear Medicine*.

[16] Chen W, Dilsizian V (2012). Molecular imaging of amyloidosis: will the heart be the next target after the brain?. *Current Cardiology Reports*.

[17] Banzo-Marraco J, Nerin-Mora E, Abos-Olivares MD (1981). Renal uptake of 67Ga-citrate in renal amyloidosis due to familiar Mediterranean fever. *European Journal of Nuclear Medicine*.

[18] Waxman AD (1982). Scintigraphic evaluation of diffuse hepatic disease. *Seminars in Nuclear Medicine*.

[19] Moyle JW, Spies SM (1980). Bone scan in a case of amyloidosis. *Clinical Nuclear Medicine*.

[20] Janssen S, Piers DA, Van Rijswijk MH, Meijer S, Mandema E (1990). Soft-tissue uptake of 99mTc-diphosphonate and 99mTc-pyrophosphate in amyloidosis. *European Journal of Nuclear Medicine*.

[21] Wall JS, Richey T, Stuckey A (2011). In vivo molecular imaging of peripheral amyloidosis using heparin-binding peptides. *Proceedings of the National Academy of Sciences of the United States of America*.

[22] Berk F, Demir H, Hacihanefioglu A (2002). Hepatic and splenic uptake of Tc-99m HDP in multiple myeloma: additional findings on Tc-99m MIBI and Tc-99m sulfur colloid images. *Annals of Nuclear Medicine*.

